# Relationship between *KCNQ1* (*LQT1*) and *KCNH2* (*LQT2*) gene mutations and sudden death during illegal drug use

**DOI:** 10.1038/s41598-018-26723-8

**Published:** 2018-05-31

**Authors:** Sayaka Nagasawa, Hisako Saitoh, Shiori Kasahara, Fumiko Chiba, Suguru Torimitsu, Hiroko Abe, Daisuke Yajima, Hirotaro Iwase

**Affiliations:** 1 Department of Legal Medicine, Graduate School of Medicine, Chiba University, 1-8-1 Inohana, Chuo-ku, Chiba City, Chiba 260-8670 Japan; 2 Department of Forensic Medicine, Graduate School of Medicine, The University of Tokyo, 7-3-1 Hongo, Bunkyo-ku, Tokyo 113-0033 Japan; 3 Department of Forensic Medicine, Graduate School of Medicine, International University of Health and Welfare, 4-3 Kozunomori, Narita City, Chiba 286-8686 Japan

## Abstract

Long QT syndrome (LQTS), a congenital genetic disorder, can cause torsades de pointes (TdP), and lethal cardiac arrhythmia may result from ingestion of cardiotoxic drugs. Methamphetamine (MP) and new psychoactive substances (NPSs) can trigger TdP due to QT prolongation, leading to sudden death. We therefore analysed variations in the LQTS-associated genes *KCNQ1* (*LQT1*) and *KCNH2* (*LQT2*) using cardiac blood and myocardial tissue from subjects having died suddenly during MP or NPS use to investigate the relationship between congenital genetic abnormalities and sudden death during illegal drug use. We amplified and sequenced all exons of these genes using samples from 20 subjects, half of whom had died taking MP and half after using NPSs. G643S, a *KCNQ1* missense polymorphism, was significantly more common among sudden deaths involving NPSs (6 subjects) than those involving MP (1 subject) and healthy Japanese subjects (P = 0.001). Notably, synthetic cathinones were detected in 2 of 3 cases involving G643S carriers. Previous functional analyses have indicated that the G643S polymorphism in the *KCNQ1* potassium channel gene causes mild I_Ks_ channel dysfunction. Our data suggest that use of NPSs, particularly synthetic cathinones, is associated with elevated risk of serious cardiac arrhythmia and sudden death for subjects carrying *KCNQ1* G643S.

## Introduction

Methamphetamine (MP) has a high potential for abuse and MP addiction has become a serious social problem worldwide^1,2^. It has been reported that in Japan, more than 10,000 people have been arrested for MP abuse per year over recent decades^3^. In addition, new psychoactive substances (NPSs) dubbed “legal highs”, “designer drugs”, and “research chemicals” have recently emerged as an important social concern in Japan despite the implementation of a new blanket dangerous substance scheduling system in 2013^4,5^. An increasing number of clinical and post-mortem studies have associated MP use with angina, tachycardia, hypertension, myocarditis, dilated cardiomyopathy, arrhythmia, and sudden death^6,7^. QT prolongation leading to fatal arrhythmia has been implicated in sudden death during MP use, and this drug has also been associated with development of cardiomyopathic states, including torsades de pointes (TdP)^8^. Similarly, various NPSs have been reported to cause TdP, which may be involved in the occurrence of sudden death during the use of such drugs^9–11^.

Long QT syndrome (LQTS) is a potentially life-threatening arrhythmic cardiac disease characterized by the presence of a prolonged QT interval on an electrocardiogram. LQTS is associated with TdP, a type of ventricular tachycardia, which may degenerate into ventricular fibrillation and cause sudden cardiac death. LQTS can be divided into congenital and secondary forms. The former include clearly hereditary cases, as well as idiopathic LQTS with unclear genetic components^12–14^. In contrast, the latter can be caused by a variety of factors, such as drugs or electrolyte abnormalities. Recent studies have reported that mutations in genes associated with congenital LQTS may also be implicated in some apparently secondary cases^15^.

Studies to date have described 13 genes (*LQT1* to *LQT13*) associated with congenital LQTS^16^. Priori *et al*. reported that carriers of certain *LQT* gene polymorphisms are at risk of developing TdP unexpectedly if exposed to cardiac or non-cardiac drugs that block potassium channels^17^. Although remarkable genetic heterogeneity is observed in LQTS, *KCNQ1* (*LQT1*) and *KCNH2* (*LQT2*), which encode potassium channel proteins, and *SCN5A* (*LQT3*), encoding a sodium channel protein, are foremost in importance, in that more than 90% of LQTS patients with identified mutations carry variants in these genes^18^. In 2014, Kamei *et al*. reported that mutations of *KCNQ1* and *KCNH2* are significantly more common among patients having died suddenly during therapy with antipsychotic drugs with QT prolonging action, and suggested that these genes are involved in sudden death associated with antipsychotic drug use^19^.

Post-mortem genetic analysis may provide insight into the pathogenesis of sudden death during illegal drug use. Considering this and the abovementioned findings, we focused on 2 genes (*KCNQ1* and *KCNH2*) associated with potassium channel abnormalities, performing sequence analysis using cardiac blood and myocardial tissue from subjects having died suddenly during MP or NPS use to investigate the relationship between congenital abnormalities in these genes and sudden death related to illegal drugs with QT prolonging effects.

## Results

We screened for mutations in *KCNQ1* and *KCNH2* in 3 groups: 10 cases of sudden death involving MP use (male/female ratio: 10:0; mean age: 51.60 years), 10 cases involving NPS use (male/female ratio: 9:1; mean age: 32.90 years), and a control group comprising 100 Japanese individuals without LQTS. Although no electrocardiogram results were available for any of the subjects, to the best of our knowledge, none of the participants had been previously diagnosed with heart conditions or had a family history of such conditions and/or sudden death. DNA extracted from cardiac blood and myocardial tissue returned the same results in all cases. Table 1 summarises the *KCNQ1* and *KCNH2* polymorphisms detected. Eight single nucleotide polymorphisms were identified, 3 in *KCNQ1* and 5 in *KCNH2*. Two of these were missense polymorphisms [1 in *KCNQ1* (G643S) and 1 in *KCNH2* (K897T)], and 6 were silent polymorphisms (2 in *KCNQ1* and 4 in *KCNH2*). Both G643S and K897T were detected in 10% (1/10) of the subjects in the MP group. In the NPS group, these polymorphisms were detected in 60% (6/10) and 10% (1/10) of subjects, respectively. In contrast, they were found to be carried by 11% and 6%, respectively, of the Japanese control group (n = 100)^19^. Thus, the frequency of the G643S polymorphism differed significantly between the NPS, MP, and control groups (P = 0.001), whereas that of the K897T variant did not (P > 0.397). Drugs were detected in the bodies of 3 of the 6 G643S carriers having suddenly died during NPS use; synthetic cathinones were used in 2 of these 3 cases.

**Table 1:** Summary of polymorphisms detected in the present study.

Gene	Nucleotide change	Amino acid change	Position within protein	Exon	Number of carriers		Frequency in control group	P-value
					NPS (n = 10)	MP (n = 10)		
*KCNQ1* (*LQT1*)	C435T	I145I	S1-S2	2	1 (Case N-9)	1 (Case M-2)	0.11	0.9919
	G1638A	S546S	C-terminus	13	1 (Case N-5)	1 (Case M-8)	0.56	0.0009
	G1927A	G643S	C-terminus	16	6 (Case N-2, 3, 5, 7, 8, 10)	1 (Case M-9)	0.11	0.0002
*KCNH2* (*LQT2*)	T1467C	I489I	S2-S3	6	8 (Case N-2, 3, 4, 5, 6, 7, 8, 10)	6 (Case M-1, 2, 3, 5, 6, 9)	0.41	0.2952
	T1539C	F513F	S3	6	8 (Case N-2, 3, 4, 5, 6, 7, 8, 10)	6 (Case M-1, 2, 3, 5, 6, 9)	0.37	0.1613
	G1692A	L564L	S5	7	0	1 (Case M-2)	0.03	0.4151
	A2690C	K897T	C-terminus	11	1 (Case N-10)	1 (Case M-5)	0.06	0.8071
	G2732C	G911G	C-terminus	12	2 (Case N-4, 7)	1 (Case M-8)	0.27	0.4615

Nucleotide numbering begins from the ATG start codon. NCBI GenBank accession numbers: AF000571 (*KCNQ1*) and AF363636 (*KCNH2*).

The control group comprised 100 Japanese individuals without LQTS (ref.^19^).

P-values < 0.05 were considered statistically significant.

## Discussion

In post-mortem examination, it is often possible to infer or identify cause of death by performing pathological tests and/or biochemical assessments, including drug analyses. Nevertheless, in some instances, cause of death can be difficult to ascertain even when such tests are used. For deaths classified as sudden, lethal arrhythmia is considered a possible cause; however, anatomical evidence indicative of cardiac arrhythmia is hard to obtain. Hereditary myocardial ion channel disorders such as LQTS and Brugada syndrome are known to cause lethal cardiac arrhythmias, and in recent years, it has become apparent that certain drugs can induce acquired LQTS^8,10,11,16,17,20,21^. Antipsychotic drugs in particular have been shown to lead to LQTS by blocking myocardial K^+^ channels, and *KCNQ1* mutations have been detected significantly more frequently among patients having died during use of such drugs^8,10,11,19,21–23^. We therefore hypothesized that asymptomatic individuals possessing *LQT* gene polymorphisms associated with K^+^ channel dysfunction face increased risk of serious cardiac arrhythmia induced by illegal drug use. To test this, we analysed *KCNQ1* and *KCNH2* variants among cases of sudden death involving illegal drug use. To our knowledge, this is the first post-mortem genetic analysis of individuals having died during illegal drug use. We believe that investigating the effects of carrying certain genetic polymorphisms on reactions to drug use may identify previously unknown drug mechanisms of action and contribute to more in-depth examinations of cause of death.

In the present study, 2 *KCNQ1* and *KCNH2* missense mutations (G643S and K897T, respectively) that have previously been reported to be involved in K^+^ channel dysfunction^24–27^ were detected in cases involving MP and NPS use. Prior functional analysis has shown that the G643S polymorphism results in a 30% reduction in the slow delayed rectifier K^+^ current (I_Ks_)^25^. This dysfunction is thought to predispose carriers to life-threatening arrhythmias in the presence of precipitating factors, such as hypokalaemia or bradyarrhythmia^25^. In the current study, the G643S mutation was detected in 6 cases (60%) in the NPS group and in 1 case (10%) in the MP group. The control group of 100 Japanese people without LQTS used in the present investigation derived from earlier research performed by Kamei *et al*.^19^. In this same work, these authors performed PCR-restriction fragment length polymorphism analysis of a further 281 Japanese individuals with no history of cardiac disease to add to the data from the initial 100 control subjects. They reported the same G643S polymorphism frequency (11%) in both the original (n = 100) and combined (n = 381) control groups^19^. In addition, Ackerman *et al*. used a combination of PCR, denaturing high-performance liquid chromatography, and automated DNA sequencing method to detect the G643S polymorphism in 110 healthy Japanese individuals and 134 Asians (60 Japanese, 56 Chinese, and 18 Filipino subjects), recording frequencies of 9% and 6%, respectively^24^.

Our results suggest that carriage of the G643S polymorphism by individuals having died of unidentifiable causes suddenly and unexpectedly while using NPSs is unusually common compared with the general Japanese population. In addition, this variant was present at a higher rate among cases of sudden death involving NPSs than those in which MP use had been recorded. Indeed, its frequency in the latter was almost the same as that among healthy Japanese individuals. No genetic mutations were present at a significant frequency in the MP group, even when cases were sorted by MP concentration. This observation suggests that despite the QT prolonging effect of MP use, the G643S mutation is not involved in MP-associated sudden death, regardless of the dose taken.

The lethal doses of many NPSs remain unknown, and the relationship between drug concentration and cause of death is unclear. In addition, identification of such drugs itself is difficult, and some substances cannot be detected following use. In this study, αPVP, αPHP, αPHPP, XLR-11, UR-144, EAM-2201, and 5-fluoro-AB-PINACA were detected in cases of sudden death during NPS use. αPVP, αPHP, and αPHPP are classified as synthetic cathinones, and their action is similar to that of MP. Seizures and cardiac arrhythmias have been reported to be the major symptoms of clinical synthetic cathinone poisoning^10^. In the present study, synthetic cathinones were involved in 2 of the 3 cases in which NPSs were detected in individuals carrying the G643S mutation. In the single case (N-1) involving synthetic cathinone use in which no genetic mutation was identified, the concentration of αPVP measured was considerably higher than that in other cases associated with use of this drug (N-8 and N-10). In the few reports of fatal αPVP intoxication, toxicology tests have revealed concentrations in post-mortem blood samples of 0.4–0.9 μg/mL^28,29^. In subject N-1, αPVP was present at 1.4 µg/mL in the cardiac blood, a high concentration even taking into account post-mortem redistribution. Therefore, we considered that cardiac arrhythmia, due to the action of αPVP, occurred regardless of the absence of G643S. Blood concentrations of αPHP and αPHPP in deaths involving these drugs have not previously been reported; therefore, the relationship between their concentrations and cause of death could not be examined in the present work.

These results suggest that the G643S mutation is related to sudden death during NPS use but not that during MP use, despite the similar structures and QT prolongation effects of some of these drugs. XPL-11, UR-144, 5-fluoro-AB-PINACA, and EAM-2201 are all cannabinoid receptor agonists, but QT prolongation by these drugs has not been reported to date. In addition, of the cannabinoid agonist-associated cases examined here, all involved low drug concentrations. However, subject N-2, whose blood contained EAM-2201, did carry the G643S mutation. Nevertheless, as EAM-2201 concentrations and the effects of EAM-2201 toxicity in cases of sudden death have not been previously described, the relationship between the concentration detected in this subject and their cause of death could not be investigated. There were 3 deceased subjects (N-3, N-5, and N-7) in the current work who carried the G643S polymorphism, but in whom no drug was detected. For cases N-3 and N-5, police are currently analysing the drugs found on site, and pentylone and nitrites appear to have been involved, respectively. Pentylone has been reported to exert a QT prolonging action, and it is possible that genetic polymorphisms are related to sudden death during its use^30^. No drugs were detected in subject N-7 by the police department, but a security camera recorded this individual apparently inhaling a herbal drug substance immediately prior to their death. Thus, the possibility that the subjects in this study used drugs that cannot be detected after death, and that such substances had QT prolonging effects cannot be ruled out.

In this study, the *KCNH2* K897T mutation was detected in 10% of subjects (1/10) in both the NPS and MP group. Kamei *et al*. reported that 9.2% of the healthy Japanese subjects that they examined using a PCR-based method carried this variant^19^. To date, no significant variation from the frequency of this polymorphism in the general Japanese population has been observed in particular groups of subjects in this country^9^. Carriage of this variant is known to differ according to ethnicity, with estimates of its frequency among Caucasians being as high as 33%. In contrast, its frequency among Asians is thought to be around 7.5%^24^. Some reports have suggested that this polymorphism may be involved in LQTS^26,27^. For instance, a previous functional analysis indicated that the K897T form of KCNH2 results in slightly lower current density than the wild type protein^27^. This investigation also found that the small decreases in current density attributable to this polymorphism are unlikely to cause disease on their own, but that this variant can reinforce the effects of reductions in the rapid delayed rectifier K^+^ (I_Kr_) current caused by certain factors, such as QT-prolonging drugs or co-inheritance of LQTS-associated polymorphisms. In the current study, the *KCNH2* K897T mutation was carried by a single NPS user, who also harboured the *KCNQ1* G643S polymorphism. Therefore, it was not possible to establish the involvement of K897T in sudden death during illegal drug use.

Together with *KCNQ1* and *KCNH2*, the sodium channel gene *SCN5A* is one of the most frequently mutated genes in LQTS; however, we did not test for *SCN5A* variants in the present study. Moreover, given the lack of electrocardiogram results for any of the subjects included, it would be important to exclude the possibility that additional mutations in other genes involved in channelopathies were present. However, it was not possible to address this point in the current work. Consequently, we were unable to establish whether the G643S polymorphism is the only factor impacting sudden death during NPS use.

## Conclusion

The number of post-mortems performed in connection with NPS-related fatalities has been increasing in recent years. However, the properties of many substances termed “NPSs” remain unknown, such as their structure, mechanism of action, and toxicological characteristics, and methods for their detection have not been established. In this study, the G643S mutation in the *KCNQ1* gene was detected significantly more frequently among sudden deaths involving NPS use, but its relationship with the effects of various drug types remains unclear. However, this variant was significantly more common among individuals having succumbed while using NPSs (particularly synthetic cathinones, notably αPVP) than those having died suddenly during use of MP, which exerts QT prolongation effects similar to those of certain NPSs. The present data suggest that use of NPSs, synthetic cathinones in particular, confers an elevated risk of serious cardiac arrhythmia and sudden death for *KCNQ1* G643S carriers. In the future, it will be necessary to investigate the mechanisms of toxicity of each NPS and the symptoms of intoxication with such drugs, and integrate these findings with analyses of other channel abnormality-associated genes, including additional *LQT* genes.

## Methods

### Sample collection

All procedures involving human participants were accordance with the 1964 Helsinki declaration and its later amendments or comparable ethical standards, and approved by the Sciences Ethics Committee of Chiba University. As part of our routine work, an informed consent paper was obtained from the immediate family members of deceased before starting autopsy.

We examined 20 cadavers in total, for which no obvious cause of death other than the presence of drugs had been identified: 10 cases of sudden death during use of NPSs, and 10 cases of sudden death during use of MP. DNA samples were extracted from myocardial tissue and cardiac blood samples. The specific details of each subject are given in Tables 2 and 3. We used previously reported gene mutation frequencies among 100 Japanese people without LQTS as a control, and the primers and PCR method described in the same report^19^.

**Table 2:** Details of each subject in the NPS group.

Number	Age (years)/sex	Circumstances of death	Drug detected (µg/mL)
N-1	40/Male	Subject was found deceased in a bathtub in a love hotel.	αPVP (HB: 1.4), UR-144 (HB: 0.003), XLR-11 (HB: 0.002)
N-2	19/Male	Subject was found deceased in bed at his home; 14 different dangerous drugs were discovered on the premises.	EAM-2201 (HB: 0.49)
N-3	36/Male	Subject was found deceased in bed at his home. Dangerous drug packaging was discovered on the premises.	No substance detected
N-4	27/Female	Subject was found deceased in bed at her home. Packaging from 5 different types of dangerous drugs was discovered on the premises.	No substance detected
N-5	51/Male	Subject suddenly collapsed after engaging in sexual intercourse while using a dangerous drug substance he concocted in his room.	No substance detected
N-6	23/Male	Subject was found dead in his car, in which an empty dangerous drug package was found.	5-Fluoro-AB-PINACA (FB: < 0.001)
N-7	27/Male	Subject was found deceased in a manga (comic book) cafe. A security recording suggested inhalation of cannabis. Dangerous drugs were discovered in the subject’s bag.	No substance detected
N-8	28/Male	Subject was found half-naked and collapsed in a corridor in his home. An empty dangerous drug package was found on the premises.	αPVP (FB: 0.070)
N-9	42/Male	Subject was found deceased in his home. Mild decomposition had occurred. An empty dangerous drug package was found.	No substance detected
N-10	36/Male	Subject was found deceased in his home. An empty dangerous drug package was found on the premises.	αPVP (FB: 0.24), αPHP (FB: 0.62), αPHPP (FB: 0.10)

Abbreviations: HB, heart blood; FB, femoral blood.

**Table 3:** Details of each subject in the MP group.

Number	Age (years)/sex	Circumstances of death	Drug detected (µg/mL)
M-1	66/Male	Subject was found convulsing in the street and died while being transported to hospital by ambulance.	MP: 0.003 µg/mL, AMP: detected
M-2	48/Male	Subject was found deceased at a capsule hotel.	MP: 0.008 µg/mL, AMP: 0.007 µg/mL
M-3	70/Male	Subject was found deceased in a corridor in his home.	MP: 0.009 µg/mL, AMP: 0.017 µg/mL
M-4	29/Male	Subject was found deceased in bed at his home.	MP: 0.026 µg/mL, AMP: 0.063 µg/mL
M-5	43/Male	Subject was found deceased in bed at his home.	MP: 0.01 µg/mL, AMP: 0.005 µg/mL
M-6	48/Male	Subject was found deceased at a hotel.	MP: 0.32 µg/mL, AMP: 0.19 µg/mL
M-7	58/Male	Subject went missing after a fight with a partner and was found deceased on a beach the next day.	MP: 0.6 µg/mL, AMP: 0.1 µg/mL
M-8	53/Male	Subject was found deceased in a corridor in his home.	MP: 0.70 µg/mL, AMP: 0.44 µg/mL
M-9	53/Male	Subject collapsed while working in a rice field and died after being transported to hospital.	MP: 0.79 µg/mL, AMP: 0.033 µg/mL
M-10	48/Male	Subject died while engaging in sexual intercourse while using stimulant drugs.	MP: 8.7 µg/mL, AMP: 0.033 µg/mL

Abbreviations: MP, methamphetamine; AMP, amphetamine.

### DNA extraction

DNA was extracted from myocardial tissue and whole mononuclear cells in cardiac blood using an EZ1® DNA Investigator kit and the EZ1® Advanced XL system (QIAGEN Inc., Hilden, Germany).

### PCR and sequencing analysis

All exons of *KCNQ1* and *KCNH2* were amplified using primers and PCR conditions previously described by Kamei *et al*.^19^ (Tables 4 and 5). For exon 1 of *KCNQ1* and exons 1, 2, and 11 of *KCNH2*, touchdown PCR was used and Q-solution (QIAGEN) was included in the PCR mix. Each reaction (20 µL) contained 50 pmol each primer, 0.2 mM dNTPs, 1 × PCR buffer (Applied Biosystems, Foster City, CA, USA), and 1.25 U AmpliTaq Gold® DNA polymerase (Applied Biosystems). PCRs were carried out using a GeneAmp 9700 system (Applied Biosystems).

**Table 4:** Primer sequences and PCR conditions used for *KCNQ1* (*LQT1*) (ref.^19^).

*KCNQ1* (*LQT1*) exon	Forward (5′-3′)	Reverse (5′-3′)	Amplicon (bp)	Tm (°C)
1.1	CTCGCCTTCGCTGCAGCTC	GCGCGGGTCTAGGCTCACC	334	TD 70–60
1.2	CGCCGCGGCCCCAGTTGC	CAGAGCTCCCCCACACCAG	244	TD 70–60
2	AATGGATGACTGGGTTTTCG	TATCAGGGCAGGACCAATGT	363	60
3	TTCTCAGGGTGTCCTTCAGC	AGGGGACTCCATCTGGTAGG	334	60
4	ATCCGAGGTGTCTCCATGTC	CATCTGAGCAAGGTGGATGG	326	60
5	AGGGACACCCATGCCATC	CTGCTCCCTCCGTCCTGT	246	65
6	CTTAGGCGTCTGCACAGGAG	GCACAGGTTTGTGGACAGAG	359	65
7	GCTCTGTTCCTGGTGCTTTC	CGTAAGTGGGTCTGCTCACA	367	60
8	ATACCTGGCCTTCCCACAAC	AAGCAGAGTATGCCCCACAG	333	60
9	TCAAGCCTGTGACTCTGAGGT	TGACACAGGCTGTACCAAGC	388	65
10	GCTGCACAGGCACTCTGG	GAAGCTCCACCCTCTGTCTG	364	60
11	ACTGATTGTCAGGGCTGGAG	TGGGCACTAGGCGAGTAGAT	376	65
12	TCTGGAAGGATCCAGTCTGC	GCCTCCAAGGATAGGTCCTC	392	60
13	AACCAGGCTTATGCCATCAC	GGTGGTTGAGAGGCAAGAAC	362	60
14	GTCAAGCTGTCTGTCCCACA	CCCTGGCTTTCATTTCATGT	270	60
15	ACCGTACCACCCCTGGTATT	CCCCTCTTGGGAGTTGCT	393	60
16	GTTGGCACCTTCCCTTCTCT	ACTCTTGGCCTCCCCTCTC	391	60

Abbreviation: TD, touchdown.

**Table 5:** Primer sequences and PCR conditions used for *KCNH2* (*LQT2*) (ref.^19^).

*KCNH2* (*LQT2*) exon	Forward (5′-3′)	Reverse (5′-3′)	Amplicon (bp)	Tm (°C)
1	CCGCCCATGGGCTCAGG	CATCCACACTCGGAAGAAGT	144	TD 62–50
2	CGCACTCTCCTCACCGCCCC	CCCTCTTGACCCCGCCCCTG	305	TD 65–70
3	GTTCACTCCCACCTCCAAA	CATCCTGCGTGGCTTTCT	378	60
4.1	ACGACCACGTGCCTCTCCTCTC	GGGACCCACCAGCGCACGCCG	267	64
4.2	CCCTGGACGAAGTGACAGCCATGG	GGCTGGGGCGGAACGGGTCC	319	68
5	CCTCCAAGGTGAGAGGAGA	GCCTGACCACGCTGCCTCT	279	60
6	TCCTCTCCCTACACCACCTG	CCTGCTGCTGGTCATCTACA	354	62
7	AAGTTCCAGGGCCTCACTCT	TCTTGCTCATGTGCACCTTT	398	62
8	CTCTGCCACCCCACTCTTC	GTCCCTGCAGAGGCTGAC	300	64
9.1	TTCCTCTACTGCCCAGGCTA	CACCGCCCTGTACTTCATCT	378	62
9.2	AGAAGGCTCGCACCTCTTG	GGTGGGATGGTGGAGTAGAG	397	62
10	ACAGCTGGAAGCAGGAGGAT	GGCTGAGCTCCCTGTCCT	275	70
11	AAGGGATGGGAAGGTCTGAG	CACTGAAAGGGCCCTGATAC	247	TD 58–70
12	CTGCAGCCAGAGAGCAGAG	CTCTGTTTCCCACAGACACG	379	50
13	GCCCTCTCCCTCTACCAGAC	AGAAGAGCAGCGACACTTGC	395	65
14	GTCACGGTACATCGAGGAAG	CCCACTTCTCTGAGCATCC	250	62
15	CTCCTCCTCCATGGCCTCT	CCTTGATCCCTGGGTGAG	243	64

Abbreviation: TD, touchdown.

PCR products were sequenced using a BigDye® Terminator v1.1 cycle sequencing kit (Applied Biosystems). Each reaction (10 µL) included *KCNQ1* and *KCNH2* primers at a final concentration of 0.25 pmol/µL and took place over 25 cycles of 30 s at 96 °C, 15 s at 50 °C, and 2 min at 60 °C. The products were purified using a QuickStep 2 PCR Purification Kit (Edge Biosystems, Gaithersburg, Germany). DNA sequencing was then performed on an ABI 3500 Genetic Analyzer (Applied Biosystems), the results of which were analysed using DNASIS Pro software (Hitachi Software Engineering Co., Ltd., Kanagawa, Japan).

### Statistical analysis

Statistical significance was evaluated using Fisher’s exact test and Fisher’s chi-squared test. P-values < 0.05 were considered statistically significant.

### Data availability

All of the data analysed during this study are included in this published article.
